# Complete anastomotic stenosis treated by combined stricturotomy using two colonoscopes

**DOI:** 10.1055/a-2127-4810

**Published:** 2023-08-30

**Authors:** Jiancong Hu, Qinghua Zhong, Dezheng Lin, Mingli Su, Xuefeng Guo

**Affiliations:** 1 Department of Endoscopy, Yuexi Hospital of the Sixth Affiliated Hospital, Sun Yat-sen University, Xinyi, Guangdong, P. R. China; 2 Department of Endoscopic Surgery, The Sixth Affiliated Hospital, Sun Yat-sen University, Guangzhou, Guangdong, P. R. China; 3 Guangdong Provincial Key Laboratory of Colorectal and Pelvic Floor Diseases, The Sixth Affiliated Hospital, Sun Yat-sen University, Guangzhou, Guangdong, P. R. China

Patients with postoperative benign anastomotic stricture after treatment for colorectal cancer can be treated successfully by endoscopic stricturotomy 1 2 . However, the treatment of complete anastomotic stenosis is difficult due to the absence of the intestinal canal. Here, we present a case of complete anastomotic stenosis treated by combined stricturotomy with two colonoscopes.

A 61-year-old man with a history of radical resection of rectal cancer and transverse colostomy 1 year previously was admitted to our hospital. Colonoscopy showed complete stenosis of the colorectal anastomosis ( Fig. 1 ). Using the distal colonic passage of the transverse colostomy, a combined stricturotomy using two colonoscopes was performed. One colonoscope (CF-H290I, Olympus) reached the oral side of the anastomosis from the transverse colostomy, while another colonoscope (PCF Q260 J, Olympus) observed from the anal side of the anastomosis. Each colonoscope was able to observe the light of the other colonoscope in the middle of the anastomotic scar. Through the oral-side colonoscope, a needle (VDK-IN, Vedkang) was inserted into the middle of the scar. The needle tip was visible from the anal side, and a circumferential incision was performed using a Hook Knife (KD620Q, Olympus), guided by the needle. After the incision, the anastomosis was recanalized, allowing the anal-side colonoscope to pass through the anastomosis into the oral-side colon ( Fig. 2 , Video 1 ). Colonography showed that the anastomosis was unobstructed and there was no leakage ( Fig. 3 ). The patient underwent colostomy closure 5 days after the stricturotomy and was discharged without any complications.

**Fig. 1:**
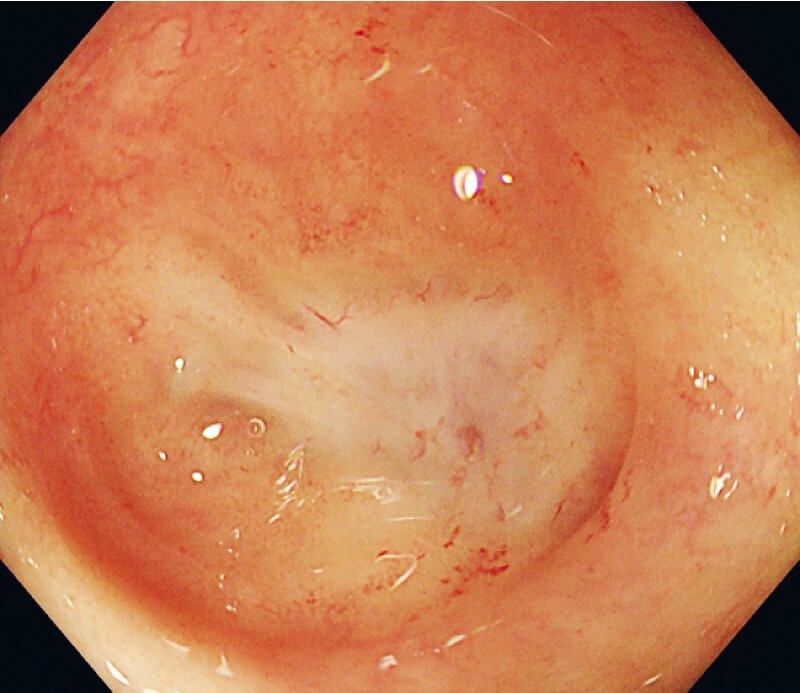
Colonoscopy showed complete stenosis of the colorectal anastomosis.

**Fig. 2:**
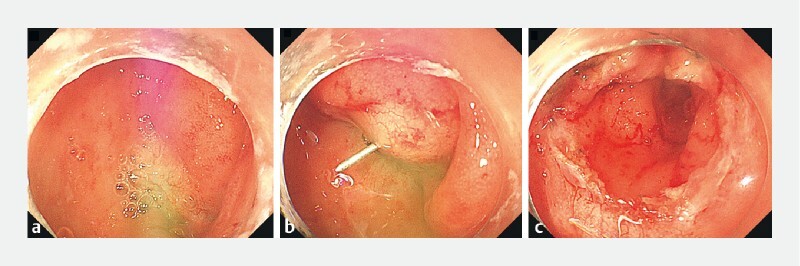
Recanalization procedure. **a** Each colonoscope was able to observe the light of the other colonoscope. **b** Circumferential incision was performed, guided by the needle. **c** The anastomosis was recanalized.

**Video 1** Complete anastomotic stenosis treated by combined stricturotomy using two colonoscopes.


**Fig. 3:**
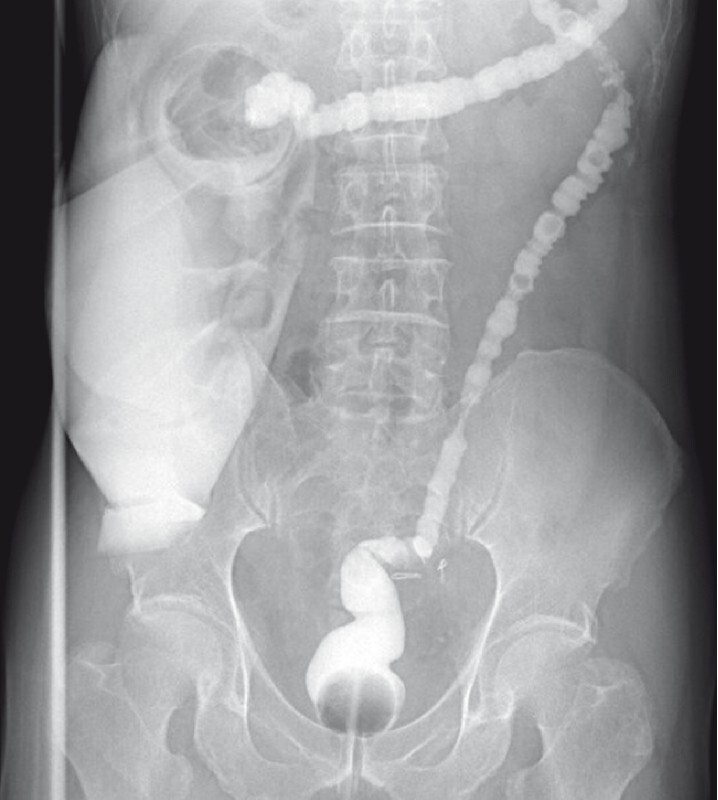
Colonography showed the anastomosis to be unobstructed.

Combined stricturotomy using two colonoscopes provides a new approach to the management of complete anastomotic stenosis.

Endoscopy_UCTN_Code_TTT_1AQ_2AF

## Notes

Endoscopy E-Videos

https://eref.thieme.de/e-videos
*E-Videos* is an open access online section of the journal *Endoscopy* , reporting on interesting cases and new techniques in gastroenterological endoscopy. All papers include a high-quality video and are published with a Creative Commons CC-BY license. Endoscopy E-Videos qualify for HINARI discounts and waivers and eligibility is automatically checked during the submission process. We grant 100% waivers to articles whose corresponding authors are based in Group A countries and 50% waivers to those who are based in Group B countries as classified by Research4Life (see: https://www.research4life.org/access/eligibility/ ). This section has its own submission website at https://mc.manuscriptcentral.com/e-videos
